# Confidentiality protections versus collaborative care in the treatment of substance use disorders

**DOI:** 10.1186/1940-0640-8-13

**Published:** 2013-08-26

**Authors:** Jennifer K Manuel, Howard Newville, Sandra E Larios, James L Sorensen

**Affiliations:** 1 University of California, San Francisco at San Francisco General Hospital, Bldg 20, Ste. 2100, Rm 2127 1001 Potrero Avenue, San Francisco, CA 94110, USA; 2 St. Luke’s-Roosevelt Hospital, Behavioral Science Research Unit, 1111 Amsterdam Avenue, New York, NY 10025, USA

**Keywords:** Electronic health records, Substance use disorders treatment, Patient confidentiality

## Abstract

Practitioners in federally-assisted substance use disorder (SUD) treatment programs are faced with increasingly complex decisions when addressing patient confidentiality issues. Recent policy changes, intended to make treatment more available and accessible, are having an impact on delivery of SUD treatment in the United States. The addition of electronic health records provides opportunity for more rapid and comprehensive communication between patients’ primary and SUD care providers while promoting a collaborative care environment. This shift toward collaborative care is complicated by the special protections that SUD documentation receives in SUD treatment programs, which vary depending on what care is provided and the setting where the patient is treated. This article explores the special protections for substance abuse documentation, discrepancies in treatment documentation, ways to deal with these issues in clinical practice, and the need for more knowledge about how to harmonize treatment in the SUD and primary care systems.

## Introduction

Increasingly, substance use assessment and treatment is occurring in health care settings such as primary care clinics, emergency departments, and trauma units. Furthermore, many health care settings have adopted or plan to adopt the use of electronic health records (EHRs), a change that can facilitate the integration of substance use disorder (SUD) treatment with other forms of health care. Thus, the landscape of SUD treatment has shifted in recent years. At the same time, current regulations for SUD treatment confidentiality have not been updated, despite the number of changes in how and where SUD is delivered. In this paper, the authors provide a summary of recent legislative and policy changes, the reasons for confidentiality protections, review the degree that substance abuse treatment documentation is protected in different environments, and suggest several directions for maintaining confidentiality while promoting coordination of care.

### The advance of electronic health records systems

The American Recovery and Reinvestment Act of 2009 (ARRA), also known as the “economic stimulus package”, provided $19.2 billion for the modernization of electronic health records [1]. The Health Information Technology for Economic and Clinical Health (HITECH Act), contained within the ARRA, also codified into law an executive order by President Bush from 2004 that created the Office of the National Coordinator for Health Information Technology (ONC) as part of the Department of Health and Human Services, which is charged with ensuring that all patients have a certified electronic health record by 2014. In addition, health care providers and hospitals who adopt a “meaningful” use of EHR will be eligible for incentive payments [2].

During this period of reform the Substance Abuse Mental Health Services Administration (SAMHSA), the agency of the Department of Health and Human Services (DHHS) responsible for the prevention and treatment of substance use disorders, has provided support for the coordination and integration of SUD treatment and healthcare. SAMHSA lists as core consensus principles in health reform to “eradicate fragmentation by requiring coordination and integration of care for physical, mental, and substance use conditions” and “adopt and fully utilize health information technology” [3] p. 4. In sum, these legislative and policy developments will increase accessibility to behavioral health services and facilitate the coordination of care for patients in health care settings with the goal of improving care for patients in the United States. Given the under-detection and under-treatment of SUDs, coordinated care environments, such as practitioners in health care settings who screen for and treat individuals who are engaging in risky or problematic substance use, can increase the detection and treatment of SUDs among patients who may not otherwise present for specialty SUD treatment [4].

### Why confidentiality laws?

*42 CFR Part 2*. The federal regulations, 42 CFR Part 2 [5,6], were developed during a time of independent SUD treatment programs, to increase treatment engagement and reduce the discrimination associated with SUD treatment. The regulations have since been updated but their purpose has remained intact: to prevent law enforcement from using substance abuse treatment patient records as a means of arresting patients [7]. Congress, wishing to make substance abuse treatment more accessible stated, “The conferees wish to stress their conviction that the strictest adherence to . . . [confidentiality] is absolutely essential to the success of all drug abuse prevention programs. Every patient and former patient must be assured that his right to privacy will be protected. Without that assurance, fear of public disclosure of drug abuse or of records that will attach for life will discourage thousands from seeking the treatment they must have if this tragic national problem is to be overcome" [8] p.33.

42 CFR Part 2 [5,6] applies confidentiality regulations to federally-assisted SUD programs, defined as an individual, entity, or unit within a general medical facility who offers or provides substance use diagnosis, treatment or referral to treatment or medical staff in a general medical care setting “whose primary function is the provision of alcohol or drug abuse diagnosis, treatment or referral for treatment” [6; 2.11]. To be consistent with the 42 CFR Part 2 language, substance use providers and settings, who meet the above criteria, will be referred to as “SUD programs” throughout our discussions of privacy laws in SUD care. “Patient” refers to “any individual who has applied for or been given diagnosis or treatment for alcohol or drug abuse at a federally assisted program and includes any individual who, after arrest on a criminal charge, is identified as an alcohol or drug abuser in order to determine that individual’s eligibility to participate in a program” [6; 2.11].

42 CFR Part 2 stipulates that the “records of the identity, diagnosis, prognosis, or treatment of any patient which are maintained in connection with the performance of any drug abuse prevention function conducted, regulated, or directly or indirectly assisted by any department or agency of the United States” [6; 2.1] may not be disclosed to others unless the patient gives prior written consent for the release of his/her information. Thus, programs meeting the 42 CFR Part 2 criteria may not share any information relating to the patient without specific patient consent. Patient consent is required for each disclosure (no blanket waivers of consent are permitted), and re-disclosure is forbidden unless a provider receives patient consent.

While the general rule is no disclosure without patient consent, the confidentiality regulations have boundaries. Disclosures to medical personnel are allowed without specific patient authorization for medical emergencies if there is an immediate threat; however this information must be documented in the patient’s records. Suspected child abuse or neglect may be reported, but restrictions still apply to the original patient records of the SUD program; in other words a report is allowed but disclosure that the patient is in a SUD program is not permitted. With the patient’s consent disclosure is authorized to a qualified service organization (QSO): This is an organization that has a written agreement (Qualified Service Organization Agreement or “QSOA”) with the SUD treatment program, stating that the QSO will be governed by the confidentiality regulations, with no disclosure without consent, and will resist judicial efforts to obtain patient records.

In recent years (2010–2011) SAMSHA set forth two sets of Frequently Asked Questions (FAQs) to further clarify how the existing legislation is applicable with the current technological advances. “Applying the Substance Abuse Confidentiality Regulations to Health Information Exchange” and “Applying the Substance Abuse Confidentiality Regulations 42 CFR Part 2” describe a variety of issues including the limitations of consent, the role of HIPPA, and the relationship between 42 CFR part 2 and state laws [9]. These FAQs are intended to clarify the 42 CFR Part 2 guidelines, however many questions remain about how to interpret the guidelines in coordinated care environments and in the context of an ever-changing digital era. Furthermore, while there has been considerable improvement in the technology for documenting treatment and the integration of SUD treatment with medical care, our current privacy laws regarding substances use disorders remain largely unchanged.

### HIPAA regulations

As years passed, additional legislation was adopted to further ensure patient privacy. In 1996, the United States Congress passed the Health Information Portability and Accountability Act (HIPAA), which contains a privacy rule that restricts the spread of Protected Health Information (PHI). HIPAA regulates PHI held by “covered entities”, such as health plans, health care clearinghouses, and health care providers. HIPAA was written for direct transfers of electronic information between providers, rather than networks streamlining information [10]. Some view HIPAA as restricting the necessary transfer of data and needing improvements to facilitate smoother communication [10-12] whereas others suggest that the HIPAA Privacy Rule should be augmented with new federal regulations [13].

Modifications have been proposed to address some of the privacy issues with 42 CFR Part 2 and HIPAA. For example, the Patient Protection Coalition, a group aiming to reform 42 CFR Part 2, has suggested permitting disclosures of demographic information, diagnosis, medications, laboratory results, and current and past treatment providers to ensure patient safety. Additionally, they support enhancing patient protections regarding discrimination based on having received SUD treatment and strengthening legal ramifications for unauthorized disclosures of information [14].

Federal privacy laws are further complicated with the advent of electronic medical records [15]. Federal privacy laws were written before the development of electronic medical records, when all records were kept in paper form, rendering some parts of these laws obsolete and other parts in opposition to the electronic changeover [16]. Various groups insist on maintaining the strict protections in 42 CFR Part 2 [17] whereas others argue for a reform of the current confidentiality protections [18].

### Integration of SUD treatment and healthcare

Increasingly, substance use treatment services are being offered in health care settings at the urging of groups such as the Institute of Medicine [19]. This shift reflects the realization that many individuals drinking or using drugs at risky levels will present for care in mainstream health care settings rather than specialty SUD programs. These patients may be in need of SUD treatment but may be unwilling or unable to seek specialty substance use care. Integrating “medically harmful substance use” information into EHRs can improve patient safety and provide better care to patients [20]. Efforts, such as the Screening and Brief Intervention and Referral to Treatment initiative funded by SAHMSA, encourage the assessment and treatment of substance use disorders in healthcare settings [21] and have been successful in reducing patients’ alcohol and drug use [22].

The integration of SUD treatment and healthcare settings can be an effective way of assessing and treating patients who may not have pursued specialty SUD care and in providing a greater continuity of care for those patients who are receiving specialty SUD treatment. However, combining these services is complicated, in part due to the greater protections offered to patients in certain settings with substance use disorders. When general health care providers assess for and provide a brief intervention in a general health care setting, the documentation of the pertinent substance use information does not fall under the SUD confidentiality regulations [23]. However, if a patient in a federally-funded healthcare setting is seen or treated by a specialty substance abuse provider whose primary job responsibility is to assess for and treat substance use patients, this patient-provider information does merit protection under the 42 CFR Part 2 confidentiality regulations. At this point, providers must document only essential information within the medical record. As Miller et al. [23] note, “The problem here is determining what is essential for primary care providers to know. Primary care providers themselves may argue that they need to know everything in order to provide the best care to the patient” (p.294).

There is ambiguity about the documentation of integrated substance abuse information in healthcare settings, with no uniform method of recording this material. For instance, when SUD treatment services were implemented into a university associated primary care clinic in New Mexico, Ernst, Miller, and Rollnick [24] reported on the lack of clarity regarding confidential protections in a primary care setting. After seeking legal counsel, the authors decided to adhere to the regulations because “the services were specific to substance abuse, and psychotherapy was provided to some patients” [24], p. 3.

The integration of SUD treatment and health care raises questions about what integration means for SUD documentation in health care settings. Integration also highlights possibly discordant regulations regarding the treatment of SUDs in substance use and health care settings. If the SUD confidentiality regulations do not apply to a general medical doctor prescribing naltrexone in a primary care setting or talking to a patient about the risks of drinking while pregnant what is the rationale for the discrepancy in protections? In Table 1 we highlight the discrepancy between the documentation of SUD information by SUD in federally assisted SUD-focused programs versus general medical providers in non-SUD settings.

**Table 1:** SUD Documentation in specialty and general medical setting

**Specialty SUD provider in federally-assisted SUD program**	**Documentation and explanation**
- Patient schedules an appointment with a SUD clinic because he is concerned about his alcohol use.	The SUD counselor documents the details of the patient’s visit in the SUD clinic notes. These notes are protected by 42 CFR Part 2 and are therefore not accessible to others outside the program (in particular the patient’s primary care provider), without a specific release of information from the patient unless there is a qualifying medical emergency or an established QSOA exists.
- Patient completes a brief assessment interview and then meets with his counselor.	
- Patient and his counselor discuss the patient’s goals for treatment, and patient is encouraged to attend weekly SUD sessions. Patient is not actively withdrawing from alcohol, so counselor decides that patient does not to attend a detoxification center.	
- Patient asks if there are medications to help him deal with cravings, but counselor indicates he does not have prescription privileges. Patient is encouraged to ask his primary care provider about the use of naltrexone.	
**Medical Provider in Emergency Room**	**Documentation**
- Patient is asked about his alcohol and drug use as part of a visit to an emergency room for a traumatic injury. Patient indicates his injury occurred while intoxicated and voices concern about drinking.	Provider documents the details of the patient’s visit, including the patient’s SUD diagnosis and history of drinking. This information is not protected by 42 CFR Part 2 because the provider’s primary function did not include the provision of substance use services nor did the interaction take place in an identified substance-focused unit.
- Provider (general medical provider) assesses substance use history and diagnoses the patient with alcohol dependence.	

### Confidentiality protections in current practice

Practitioners ask as we move toward an era where medications are increasingly being prescribed for SUD, are the current protections potentially harmful to patients? Providers of patients who are prescribed naltrexone, buprenorphine, or methadone may need this information in order to accurately treat their patients. While the 42 CFR Part 2 guidelines indicate that patient information may be disclosed in medical emergency situations, preventing a potentially fatal drug interaction does not necessitate a disclosure if there is not an immediate medical emergency [25]. A study by Walley et al. [26] found that 59% of the opioid patients on methadone maintenance therapy (MMT) were also prescribed medications with a potentially harmful interaction with their MMT. In fact, 19% of the patients were prescribed three or more medications that could potentially interact with MMT. Currently, the patient must serve as the intermediary between their SUD and healthcare providers or sign an authorization of release of information for each of their particular providers. As a result “failure to share patient-specific information across providers promotes uncoordinated and sometimes unsafe care” [27], p. 716.

Nonetheless, in a recent national survey, adults have expressed some concerns about the potential for shared health information. In this survey, 42% of participants indicated that they would feel uncomfortable if their private health information was shared with other organizations, even if any identifying information was excluded. Furthermore, 15% of participants reported that they would hide information and 33% indicated that they may hide information from their doctor if he/she shared information through an EHR [28]. Patients with substance use disorders or risky substance use may be more likely to hide substance use information out of privacy concerns and fears of stigmatization.

### Solving a clinical conundrum: maintaining confidentiality in a coordinated care environment

In 2010 an article in Alc*oholism* &*Drug Abuse Weekly*[7] asked, “Can electronic medical records and patient confidentiality coexist?” The answer appears to be “Yes, but they need to be harmonized.” People involved in this tuning process will include legislators, judges, attorneys, and insurance adjusters, but a major place where these issues will be acted out is in human service programs treating people with SUD. With that in mind, we provide some suggested actions that staff in SUD treatment programs can take to cope with these controversies.

#### Keep context in mind

For all providers and other healthcare personnel, it is vital to understand the reasons why the confidentiality laws exist and also why the context is changing. Gaining an understanding in the principles, laws, and guidelines is important. Then when the question arises about protection of confidentiality, ethical principles such as respect for persons, autonomy, justice, beneficence, and non-maleficence can be part of the decision-making process [29]. The federal confidentiality statutes need to be followed, as well as various guidelines from legislation like HIPAA and applicable state and municipal regulations.

Guidelines are available from several valuable resources. CSAT/SAMHSA has developed over 50 written Treatment Improvement Protocols. These are available online at http://www.ncbi.nlm.nih.gov/books/NBK14119/ and were written for staff in a wide variety of SUD treatment programs. CSAT has also published a number of Technical Assistance Publications (TAPS), including TAP 13, *Confidentiality of patient records for alcohol and drug treatment*[30]. TIP 40, *Clinical guidelines for the use of buprenorphine in the treatment of opioid addiction,* has specific information on the confidentiality requirements for physicians who are providing office-based opioid addiction treatment. The TIP also includes a sample consent form to release information protected by 42 CFR Part 2 [31]. Additionally the Legal Action Center (LAC) can be an excellent resource, available online at http://www.lac.org/. LAC is a non-profit law and policy organization that seeks to fight discrimination against individuals with addictions, HIV/AIDS or criminal records. They provide on-line courses available both in basic and advanced issues concerning confidentiality, as well as brochures, sample forms for physicians and treatment programs, and policy analyses, including a paper on confidentiality of alcohol and drug records in the 21st Century.

#### Consider state laws

State laws may institute additional protections on patients’ privacy, so it is important to remain informed about state confidential protections. While states may require additional protections, they may not authorize a program to disclose information that is protected by 42 CFR Part 2. Thus, state laws may provide additional protections but they may not undermine the federal protections.

#### Seek guidance

When a provider approaches an uncomfortable situation it is wise to seek guidance. Counselors, peers, clinical supervisors, physicians, and licensed professionals at the SUD treatment program are essential for support, and professional associations can be helpful. For example the NAADAC, the National Association for Addiction Professionals (http://www.naadac.org/), offers webinar trainings, one of which is Compliance with Ethical Standards for Drug, Alcohol and Addictions Counselors. We stress that the specific guidelines may be changing constantly as new information emerges. For example, earlier in this paper we mentioned that in July 2010 CSAT released a “Frequently Asked Questions” paper (http://www.samhsa.gov/HealthPrivacy/docs/EHR-FAQs.pdf). The paper was initially drafted by the LAC. Enos [32] documents the many points of controversy about this document, and a month later SAMHSA sponsored a stakeholders meeting to air different viewpoints and clarify the “FAQs”. We also emphasize the importance of seeking legal guidance, which is a vital step in situations where rules may be changing, and new legal precedents may be emerging. A treatment counselor is likely to need both clear and authoritative guidance from a legal professional.

#### Implement

At the clinic level, update guidelines for your patients, staff, and referral sources, and if guidelines do not exist, it may be wise to develop them. Examples of such guidelines are available from the CSAT TIPS and TAPS mentioned above, or they may be available locally or through the single state agency that is responsible for SUD treatment.

#### Seek feedback

Obtaining feedback from those affected is an obvious suggestion, but feedback often happens only when something has gone wrong. Encourage quality assurance studies to understand what is working and why. Encourage research to understand how serious specific issues are in your setting. As an example of the utility of research, Salomon et al. [33] surveyed psychiatric clinicians who had recently switched to an electronic health record system and found the majority opinion was that open therapeutic communications were not interfered with. On the other hand, once they were using the electronic health record system the majority was less willing to record highly confidential information. In short, the clinicians indicated that the system worked but they were more cautious than before about recording sensitive information.

#### Keep learning

At the individual level, keep up to date as guidelines change. A 2009 CSAT TIP entitled *Clinical Supervision and Professional Development of the Substance Abuse Counselor* may be helpful (TIP 52) [21]. At the program and system levels attend to the needs for training among those in your system. In addition, as the ethical standards continue to evolve in substance abuse treatment, it is essential that providers remain up to date on the latest ethical guidelines.

## Conclusions

This manuscript explored protecting confidentiality in SUD treatment programs in the light of changes that have occurred recently in the policies and regulations. Legislation has been enacted to make SUD treatment more affordable and accessible to patients in need. Treatments are increasingly being offered in health care settings such as primary care clinics, emergency, and trauma units. Technological development has fostered the development and proliferation electronic health records rather than paper charts. In this context we acknowledge the difficulty of maintaining patient confidentiality versus sharing vital information with providers in a collaborative care setting. In this paper the authors provide a summary of recent legislative and policy changes, the reasons for confidentiality protections, review the degree that substance abuse documentation is protected in different environments, and suggest several directions for maintaining confidentiality while promoting coordination of care. The field of substance abuse treatment has changed drastically since the time when the privacy laws were first developed. SUD treatment is more widely available, provided by a broad range of health care providers, and is often treated by medications. The integration of SUD care has reached patients who may not have otherwise sought treatment.

Our paper provides guidance and advice to staff in SUD treatment programs regarding how to negotiate confidentiality issues in treatment programs. The issues are changing, and the ethical principles of good health care and professional practice will be increasingly relevant in this time when the field is undergoing significant change in its clientele, location of services, and its technology. Until the field stabilizes again it will be the helping professionals in the human service settings who make the most important decisions about what is best for their clientele.

This analysis is limited by several factors that can be informed through further research. There is a need for clear ethical analyses that explore the advantages and drawbacks of topics such as protecting patient confidentiality versus promoting integrated care. It would be helpful to understand more about the extent of stigmatization of SUD and whether, over time the stigma has diminished or increased. This information can inform guidance about the extent to which confidentiality protections are needed by those receiving treatment for SUD. Additional research needs to provide information about what are the most frequently occurring and most troubling ethical confidentiality and privacy issues facing various actors in SUD treatment and prevention. In addition we need to know how these issues are being resolved in the field.

Further there is a need for concerted organizational attention to resolve an increasingly acrimonious debate about the need to keep versus revise the federal confidentiality regulations that will soon have been enacted fully 50 years ago. Our current confidentiality regulations for SUD treatment have not been updated, despite the number of changes in location and context of care programs. It may be that federal confidentiality guidelines will be rewritten to reflect the changing times, or like the U.S. Constitution, the field may look to these as enduring adages that need to be interpreted to provide more clear guidance to a rapidly evolving field. In either case, there is a need for the national action to enact administrative mechanisms that will air the many facets of the issue and then come to a resolution. With clarity at the federal level the state and local agencies will be able to attune their regulations and guidelines so that a national approach to these issues can develop.

## Competing interests

The authors declare that they have no competing interests.

## Authors’ contributions

JKM drafted *Confidentiality protections in current practice*, *Integration of SUD treatment and healthcare,* and edited the manuscript. Together, JKM and SEL drafted Table 1. HN drafted parts of *The advance of electronic health records, Why confidentiality laws* and *HIPAA regulations.* SEL drafted parts of *The advance of electronic health records, Why confidentiality laws* and *HIPAA regulations.* JLS drafted *Why Confidentiality Laws, Solving a clinical conundrum* and *Conclusions* and edited the manuscript. All authors read and approved the final manuscript.
